# Stereoselectivity of Aminoacyl-RNA Loop-Closing Ligation

**DOI:** 10.1021/jacs.4c16905

**Published:** 2025-05-29

**Authors:** Shannon Kim, Marco Todisco, Aleksandar Radakovic, Jack W. Szostak

**Affiliations:** Howard Hughes Medical Institute, Department of Chemistry, 89051 University of Chicago, Chicago, Illinois 60637, United States

## Abstract

The origin of amino acid homochirality remains an unresolved question in the origin of life. The requirement of enantiopure nucleotides for nonenzymatic RNA copying strongly suggests that the homochirality of nucleotides and RNA arose early. However, this leaves open the question of whether and how homochiral RNA subsequently imposes biological homochirality on other metabolites, including amino acids. Previous studies have reported moderate stereoselectivity for various aminoacyl-RNA transfer reactions. Here, we examine aminoacyl-RNA loop-closing ligation, a reaction that “captures” aminoacylated RNA in a stable phosphoramidate product, such that the amino acid bridges two nucleotides in the RNA backbone. We find that the rate of this reaction is much higher for RNA aminoacylated with L-amino acids than for RNA aminoacylated with D-amino acids. We present an RNA sequence that nearly exclusively captures L-amino acids in loop-closing ligation. Finally, we demonstrate that ligation of aminoacyl-L-RNA results in an inverse stereoselectivity for D-amino acids. The observed stereochemical link between D-RNA and L-amino acids in the synthesis of RNA stem-loops containing bridging amino acids constitutes a stereoselective structure-building process. We suggest that this process led to a selection for the evolution of aminoacyl-RNA synthetase ribozymes that were selective for L-amino acids, thereby setting the stage for the subsequent evolution of homochiral peptides and, ultimately, protein synthesis.

## Graphical abstract

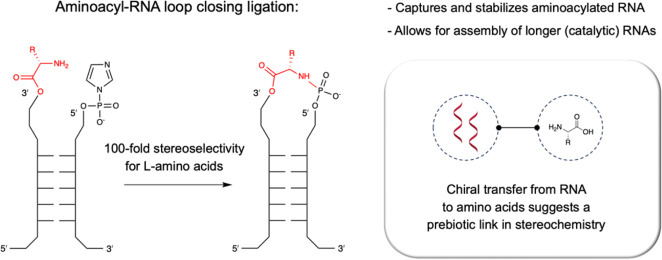

## Introduction

In all known life, nucleic acids and proteins are composed exclusively of D-nucleotides and L-amino acids, respectively. The universality of biological homochirality implies that the use of enantiopure nucleotides and amino acids was established very early in the origin of life. Experiments with model systems for nucleic acid copying and catalysis show that heterochiral components impede reactions that are fundamental for the origin of life.^1,2^ Nonenzymatic RNA template copying reactions are severely inhibited when both D- and L-nucleotides are present.^3^ Furthermore, if RNAs containing both L- and D-nucleotides were produced, the copying of such heterochiral templates would likely fail due to inconsistencies in the orientations of bound substrates.^3^ Ribozyme catalysis, in the form of a polymerase ribozyme that can synthesize homochiral RNA from mixtures of L- and D-nucleotides,^4^ may appear to offer a solution, but the ribozyme itself must start out as homochiral RNA, suggesting that homochiral components had to precede the emergence of ribozymes. Homochiral nucleotides therefore appear to be a prerequisite for the emergence of genetic self-replication. However, it remains an open question whether homochirality in other classes of biomolecules, such as amino acids and lipids, was established as a consequence of the prior homochirality of RNA. For example, if early metabolic reactions were catalyzed by a network of ribozymes, then the intrinsic stereoselectivity of macromolecular catalysis could have led to the stereoselective synthesis of chiral metabolic intermediates as well as products such as amino acids. Alternatively, the chirality of other biomolecules could, in principle, be established independently. Considerable research has gone into mechanisms of symmetry breaking^5–8^ and chiral amplification^9,10^ with regard to the origin of life. Viedma ripening, through attrition-enhanced deracemization, can result in a homochiral solid phase of conglomerate crystals from a racemic solution.^11^ The autocatalytic Soai reaction can yield near-homochiral alkylated pyrimidine 5-carbaldehydes from very slight enantiomeric imbalances in the starting material.^12^ However, neither of these processes has been shown to result in the homochiral synthesis of prebiotically relevant compounds.

A recent proposal for the origin of biological homochirality leverages the chiral-induced spin selectivity (CISS) effect to achieve enantioselective separation or crystallization on magnetic surfaces. The potential of this approach was first demonstrated by Banerjee-Ghosh et al. through the enantioselective adsorption of peptides on a magnetized surface.^13^ Tassinari et al. subsequently reported the separation of the enantiomers of the amino acids asparagine and glutamic acid by enantioselective crystallization on a gold-capped nickel surface in a magnetic field.^14^ However, the handedness of the amino acid that was enriched in a given magnetic field orientation differed depending on the amino acid side chain.^14^ Crystallization-based approaches to biological homochirality via the chiral resolution of amino acids face the issue that only four biological amino acids (asparagine, aspartic acid, glutamic acid, and threonine) have been observed to crystallize as conglomerates.^14,15^ Furthermore, conglomerate crystals of aspartic acid and glutamic acid are formed only when crystallization proceeds from solutions with molar concentrations of amino acid.^16^ Though the above studies employed nonprebiotically plausible surfaces, the use of a magnetic field to direct enantioselective crystallization has found greater prebiotic significance as a potential origin of homochirality in nucleotides. Using a magnetite surface, Ozturk et al. achieved enantioselective nucleation of the crystallization of riboseaminooxazoline (RAO), where L-/D-selectivity was dependent on the direction of magnetization.^17^ RAO is a nucleotide precursor in the cyanosulfidic prebiotic chemical network proposed by Xu et al.^18^ A process leading to homochiral RAO would lead directly to the homochiral synthesis of all downstream nucleotides and thus to homochiral RNA and DNA.

Based on the biological pairing of D-RNA and L-amino acids, it is possible that the initial selection of L-stereochemistry for amino acids may have been governed by interactions with homochiral RNA.^19,20^ Previously, Tamura and Schimmel reported a 4-fold chiral selectivity for L-amino acids in aminoacyl transfer from an RNA 5′-phosphate to the 3′-hydroxyl of an upstream oligomer in a nicked duplex.^19,21^ Wu et al. showed up to a 10-fold preference for l-alanine in interstrand aminoacyl transfer from the 5′-phosphate to the diol of RNA.^22^ Roberts et al. demonstrated 5- to 10-fold higher yields at room temperature (and 10- to 50-fold at −16 °C) for the formation of L- vs D-aminoacyl-ester RNA in a reverse loop-closing ligation beginning with the amino acid anchored to the 5′-phosphate as a phosphoramidate.^23^ On the other hand, Kenchel et al. examined the stereoselectivity of ribozyme-catalyzed self-aminoacylation and found that different ribozymes exhibited stereoselectivity for either D- or L-amino acids.^24^ We have recently described a two-step reaction system for the formation and capture of aminoacylated RNA, in which the addition of activated amino acids to RNA results in aminoacylation followed by loop-closing ligation.^25,26^ Our results suggested that this process of aminoacyl capture could be stereoselective, but in that study, we could not distinguish between selectivity at the stage of aminoacylation, ligation, or hydrolysis.

Here, we report on the stereoselectivity of loop-closing ligation with RNA that is preaminoacylated with either an L- or a D-amino acid. We do not consider the stereoselectivity of aminoacylation, as we generate aminoacylated RNA using the Flexizyme ribozyme. In the loop-closing ligation reaction, the amine of an aminoacylated RNA attacks an activated 5′-phosphate to form a phosphoramidate linkage. We find a widely varying stereoselectivity in favor of RNA aminoacylated with L-amino acids for this reaction, with the magnitude of the selectivity depending on the RNA sequence and structure. With all RNA architectures, we observe a preference for the ligation of L-aminoacylated RNA, but with one structure in particular, we observe an almost 200-fold faster rate of loop-closing ligation when the RNA is aminoacylated with an L-amino acid, highlighting the impact of the RNA tertiary structure on the stereoselectivity of this reaction. When we carry out aminoacyl loop-closing ligation using L-RNA, we observe a reversed stereoselectivity in favor of D-amino acids, demonstrating that the handedness of RNA is the chirality-determining factor in this system. Our results strengthen the hypothesis that stereoselection of L-amino acids occurred through chiral transfer from RNA, and more specifically, that the biological pairing of L-amino acids with D-RNA resulted from the stereochemical properties of aminoacylated RNA.

## Results

Our previous studies of the aminoacyl capture reaction hinted at stereoselectivity at some stage of the process, which prompted us to investigate the stereoselectivity of this reaction in greater detail. For our experiments, we preaminoacylated the 2′(3′)-diol of an “acceptor” RNA oligonucleotide using the Flexizyme, an aminoacyl-RNA synthetase ribozyme,^27^ which allows for RNA aminoacylation with both L- and D-amino acids.^28^ We then incubated the aminoacylated acceptor strand with a “capture” strand carrying a 5′-phosphorimidazolide moiety. The acceptor and capture oligonucleotides anneal to form a duplex stem with noncomplementary overhangs (Scheme 1A), such that a successful ligation reaction produces an amino acid-bridged RNA stem-loop. We studied three different RNA architectures and measured the rate and stereoselectivity of loop-closing ligation in each construct. The constructs RNA 1 and RNA 2 (Scheme 1B) were adapted from the stem-loops of the Flexizyme ribozyme. RNA architecture 3 (Scheme 1B) was identified through a deep-sequencing screen of 3′-overhang sequences that facilitate self-glycylation and ligation.^26^ RNA 3 undergoes efficient aminoacylation and aminoacyl-RNA ligation in the presence of glycylimidazolide, but these reactions occur at the internal 2′-hydroxyl of the penultimate nucleotide. In the present study, in which aminoacylation is achieved using the Flexizyme, all aminoacylation and aminoacyl ligation occur at the 2′,3′-diol of the 3′ terminal nucleotide. Because of the compact T-loop-like structure of RNA 3 that optimally positions the penultimate 2′-OH for glycylation and loop-closing ligation, the ligation rates we observe with 2′,3′-aminoacylated RNA 3 are much slower than the ligation rates that we previously observed for glycylated RNA 3.

**Scheme 1:**
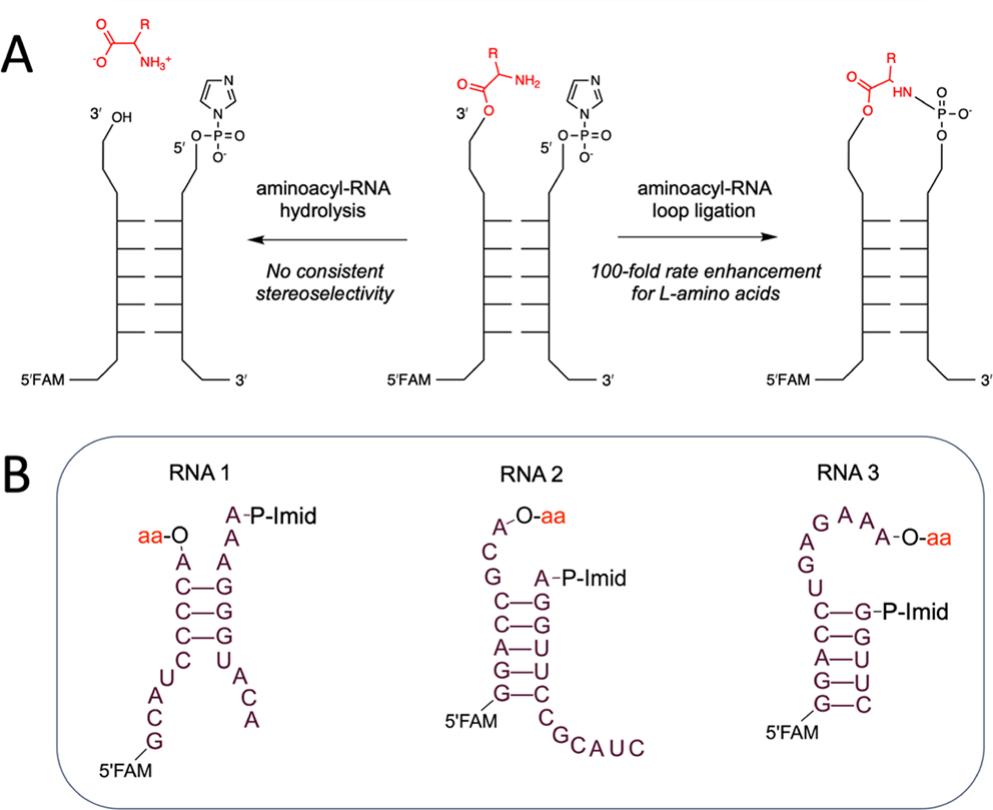
Outline of the Reactions and RNA Architectures Studied in this Work.^a^

a (A) Diagram of the reactions of aminoacylated RNA annealed to a capture strand with an activated 5′-phosphate (center). Left: hydrolysis of the aminoacyl ester. Right: ligation of the aminoacylated strand to the capture strand. Relative rates for L- over D-aminoacylated RNA reach the order of 10^2^ while hydrolysis of the aminoacyl ester occurs without a consistent stereochemical bias. (B) Secondary structures of the three RNA architectures used in this study. P-Imid indicates activation of the nucleotide 5′-phosphate with imidazole.

As a preliminary test, we examined the aminoacyl-RNA ligation of architecture 1 with l- and d-alanine. We observed greater loop-closing ligation with l-alanine, with 35% of the l-alanylated acceptor strand ligating after 1 h, as opposed to <1% ligation of d-alanylated acceptor (Figure 1A). To test whether the observed stereoselectivity might simply reflect stereoselective aminoacyl-RNA hydrolysis, we measured the rate of hydrolysis of the aminoacyl-RNA ester when the aminoacylated acceptor strand was annealed with a capture strand with an unactivated 5′-phosphate, so that ligation could not occur (we refer to this reaction condition as “duplex hydrolysis”). To account for any unanticipated effects of the capture strand on the stability of the aminoacyl-RNA, we also measured the hydrolysis rate in the absence of the capture strand (which we refer to as “single-stranded hydrolysis”). In the duplex condition, we observed that l-alanylated RNA hydrolyzed approximately 1.2 times faster than d-alanylated RNA (*p* < 0.01, Figure 1B). In the single-stranded condition, the two stereoisomers hydrolyzed at approximately the same rate (Figure 1C), suggesting that the complementary strand may have a modest influence on the hydrolytic lability of the aminoacyl ester. When we performed these reactions with leucine, lysine, and proline, we observed that the two stereoisomers hydrolyzed at comparable rates (within a factor of 0.7- to 1.2-fold, Tables S1 and S2), thus excluding preferential hydrolysis as the explanation for the stereoselective formation of the loop-closed product.

**Figure 1:**
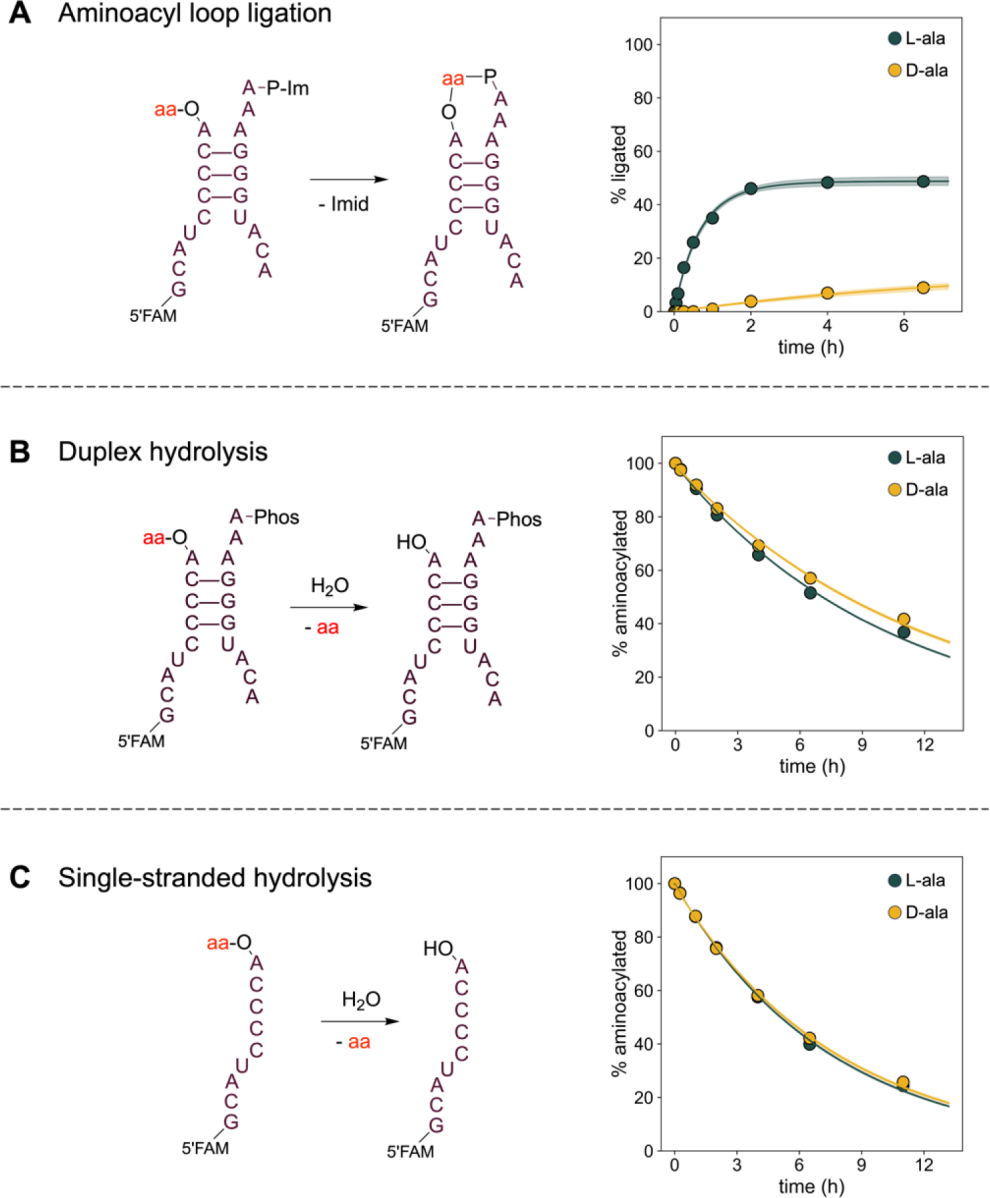
Reaction Schemes and Time Courses for aminoacyl-RNA loop-closing ligation and hydrolysis. (**A**) Left: reaction scheme for aminoacyl-RNA loop-closing ligation using an imidazole-activated capture strand. Right: time course of the ligation reaction. (**B, C**) Left: reaction schemes for aminoacyl-RNA hydrolysis under duplex and single-stranded conditions. Right: time courses of the hydrolysis reaction. Reaction rates are presented in Tables 1, S1 and S2, and gel images are shown in Figure S1. All reactions were conducted at 0 °C in 5 mM MgCl_2_, 100 μM Na_2_EDTA, 100 mM imidazole, pH 8.0, and 5 μM RNA oligonucleotides. Shaded regions envelope the 95% prediction interval as determined from the kinetic model.

To explore the generality of the stereoselectivity of aminoacyl-RNA loop-closing ligation, we performed the ligation and hydrolysis reactions with three additional amino acids: proline, lysine, and leucine, chosen to represent a diversity of side chains. For all four amino acids, loop-closing ligation plateaued more quickly and led to higher yields with the l-enantiomer than with the D-enantiomer (Figure 2). In contrast, there were minimal differences in the rate of hydrolysis between the two stereoisomers of the aminoacylated RNAs (Tables S1 and S2). To quantitatively compare the loop-closing ligation kinetics with the two enantiomers of the amino acids, we kinetically modeled the ligation reactions using a set of differential equations to account for the aminoacyl-RNA hydrolysis (as determined from our experiments measuring hydrolysis in a duplex where the 5′-phosphorimidazolide is replaced with a 5′-phosphate) and 5′-phosphorimidazolide hydrolysis (as determined from our recent work).^29^ We also accounted for the reshuffling of oligonucleotides in complexes,^30^ since loop-closing ligation can only occur when both aminoacyl and imidazole groups are present on the oligonucleotide termini. In some cases, the maximum ligation yield was lower than expected based on the rate of aminoacyl-RNA hydrolysis (notably with architecture 3, alanine, and leucine). To account for this, we allowed the model to determine the initial percentage of imidazole activation as a free parameter shared between the L- and D-aminoacylated constructs, since all pairwise experiments were conducted simultaneously. Upon fitting our traces^31^ to extract kinetic constants, we consistently observed a rate enhancement for the ligation of the L- over the d-enantiomer, with the enhancements ranging from 5-fold for l-proline to >100-fold for l-lysine and l-leucine (Figure 2, S3). In the case of lysine, both the ε- and α-amino groups were potential nucleophiles. We observed similar reactivity for ε-acetyl Lys and Lys, but no reactivity with α-acetyl Lys, indicating that lysyl-RNA ligation occurs exclusively through the α-amine of lysine (Figure S2).

**Figure 2:**
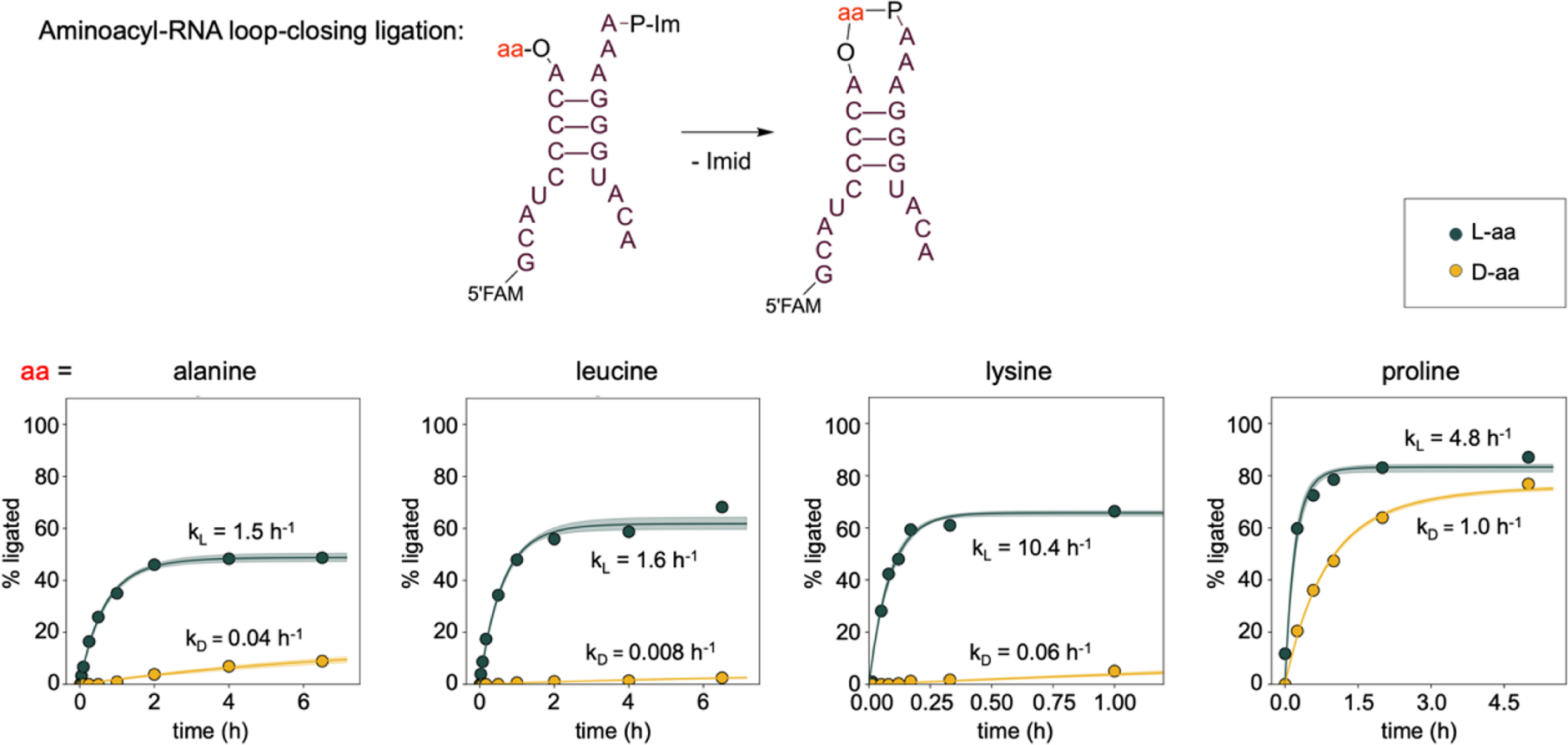
Aminoacyl-RNA loop-closing ligation for RNA architecture 1 with four different amino acids. Top: RNA architecture. Bottom: time course of loop-closing ligations. The % ligated product was determined as in Figure 1 from gel images. All reactions were conducted in three technical replicates at 0 °C in 5 mM MgCl_2_, 100 μM Na_2_EDTA, and 100 mM imidazole, pH 8.0, with 5 μM RNA oligonucleotides. Shaded regions envelope the 95% prediction interval as determined from the kinetic model. See also Figure S3 for hydrolysis timecourses.

The loop-closing reaction with RNA architecture 1 is clearly stereoselective for all four amino acids tested. To determine whether the sequence and structure of the RNA play a role in determining the stereoselectivity of the reaction, we repeated the ligation and hydrolysis experiments with two additional RNA architectures (Figures 3, S4 and S5). In these experiments, we continued to observe a stereoselective link between D-RNA and L-amino acids (Figure 3). However, the magnitude of stereoselectivity depended strongly on the specific overhang, with RNA 1 exhibiting the greatest stereoselective effect (on the order of 10^1^–10^2^). For RNA 2 and RNA 3, the ligation rate difference between RNA aminoacylated with L- vs D-amino acids ranged from 1.7-fold to 10-fold (Table 1). Interestingly, the rates and stereoselectivity of loop-closing ligation varied widely across different combinations of RNA architecture and amino acid, suggesting that specific interactions between the amino acid and the RNA structure influence the outcome of the reaction. Nevertheless, for every combination of overhang and amino acid (12 in total), ligation proceeded more efficiently with L-amino acids than their D-enantiomers (Figure 3). Faster rates of loop-closing ligation correlated with higher ligation yields, as expected for a process that involves a partition between highly variable ligation and relatively constant hydrolysis (Figures S3–S5). In general, the hydrolysis reaction exhibited very little stereoselectivity, with a maximum rate difference of 1.6-fold, indicating that differences in hydrolysis rates do not explain the observed differences in ligation rates (Tables S1 and S2).

**Figure 3:**
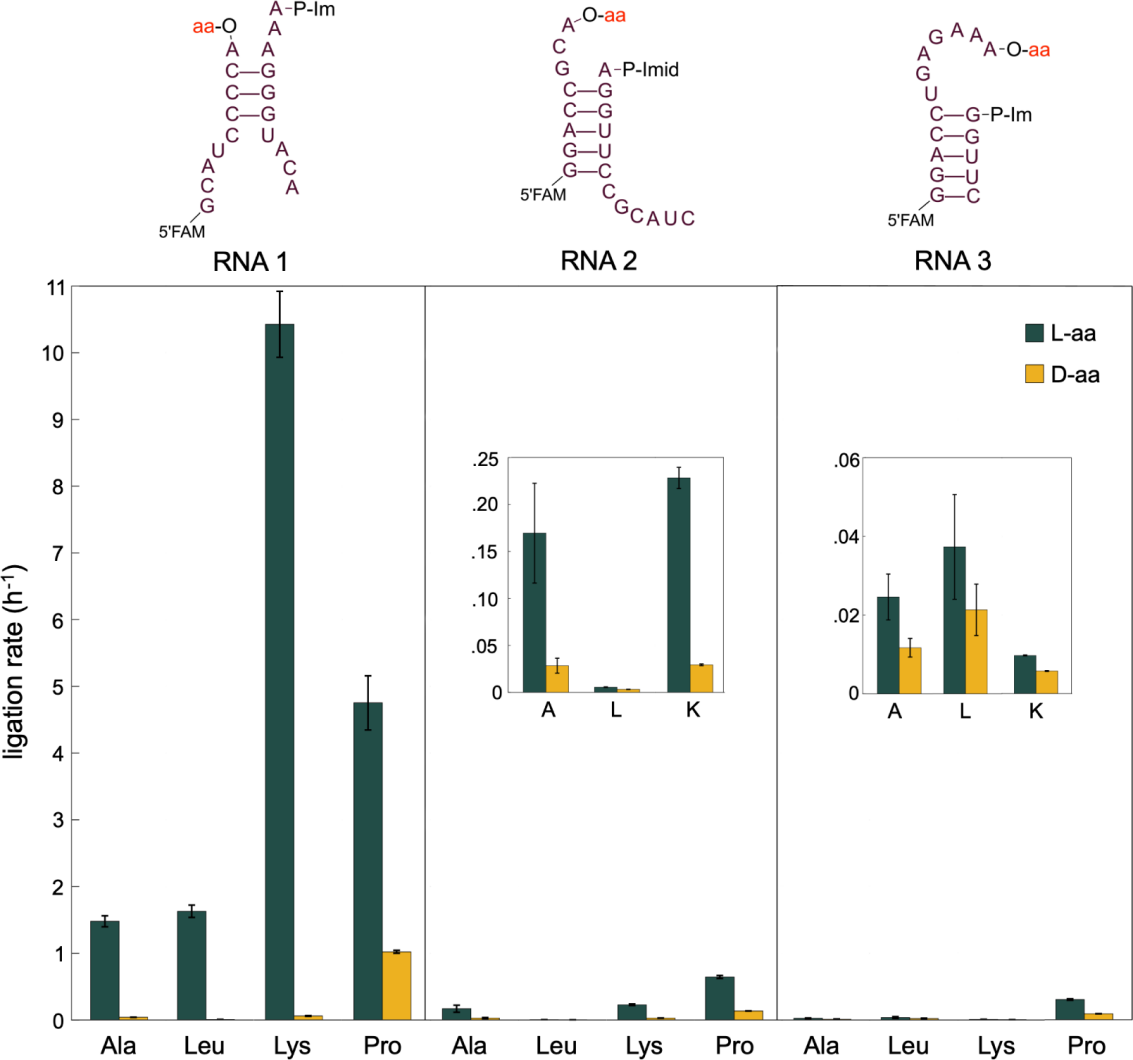
Aminoacyl-RNA ligation rates for three different RNA architectures and four different amino acids. Top: schematics for RNA architectures 1, 2, and 3. Bottom: observed rates for aminoacyl-RNA loop-closing ligation were for the three RNA architectures with the amino acids alanine, leucine, lysine, and proline. Ligation reactions were conducted in triplicate at 0 °C in 5 mM MgCl_2_, 100 μM Na_2_EDTA, 100 mM imidazole, pH 8.0, with 5 μM RNA oligonucleotides. Dark green bars: RNA aminoacylated with L-amino acids; yellow bars: RNA aminoacylated with D-amino acids. Ligation rates were derived from kinetic models using ligation yields and experimental rates for aminoacyl hydrolysis (see Kinetic Analysis section in the Materials and Methods). All L- and D- pairwise comparisons were statistically significant, with L-aminoacyl loop-closing ligation rates higher than D- in all cases. Error bars and significance were estimated as detailed in the Statistical Analysis section in the Materials and Methods.

**Table 1:** Ligation Rate Constants for the Three RNA Architectures with the Four Amino Acids: Ala, Leu, Lys, and Pro^a^

		*k*_L_ (h^–1^)	*k*_D_ (h^–1^)	*k*_L_/*k*_D_
RNA 1	Ala	1.48 ± 0.08	041 ± .003	36
	Leu	1.62 ± 0.09	0.0084 ± 0.0004	194
	Lys	10.4 ± 0.5	0.061 ± 0.006	172
	Pro	4.8 ± 0.4	1.02 ± 0.02	4.7
RNA 2	Ala	0.17 ± 0.06	0.028 ± 0.008	6.0
	Leu	0.0056 ± 0.0003	0.0033 ± 0.0002	1.7
	Lys	0.23 ± 0.01	0.029 ± 0.001	7.8
	Pro	0.64 ± 0.02	0.135 ± 0.002	4.8
RNA 3	Ala	0.025 ± 0.006	0.012 ± 0.002	2.1
	Leu	0.037 ± 0.013	0.021 ± 0.007	1.7
	Lys	0.0097 ± 0.0001	0.0058 ± 0.0001	1.7
	Pro	0.30 ± 0.01	0.091 ± 0.001	3.4

a Ligation rates were derived from kinetic models using ligation yields as a function of time and experimental rates for aminoacyl hydrolysis (see Kinetic Analysis section in the Materials and Methods). The rightmost column shows the ratio of kL/kD, and all differences between L- and D-ligation rates are statistically significant (*p* < 0.05, non-parametric test).

To determine whether the chirality of RNA is indeed responsible for the stereoselectivity of the loop-closing ligation reaction, as opposed to any unknown and uncontrolled variable such as the presence of chiral contaminants, we repeated the ligation and hydrolysis reactions using synthetic L-RNA rather than canonical D-RNA. L-RNA was prepared by solid-phase synthesis using L-nucleotide phosphoramidites and aminoacylated using an L-RNA version of the Flexizyme ribozyme. We observed that inversion of the RNA chirality from D- to L- resulted in loop-closing ligation favoring D-amino acids for all three RNA architectures with the amino acids proline and lysine (Figures 4, S6). Differences in the reaction rates between D- and L-RNA are most likely due to differences in the purity of synthetic substrates and minor variations in the experimental conditions. Importantly, the overall pattern of the ligation reaction rates was conserved but reversed between D- and L-RNA. Notably, we observed highly stereoselective ligation with L-RNA 1 and lysine (130-fold rate enhancement with d-lysine over l-lysine, Figures 4, S6 and Table 2), which mirrors the result with D-RNA 1 (170-fold rate enhancement with l-lysine over d-lysine).

**Figure 4:**
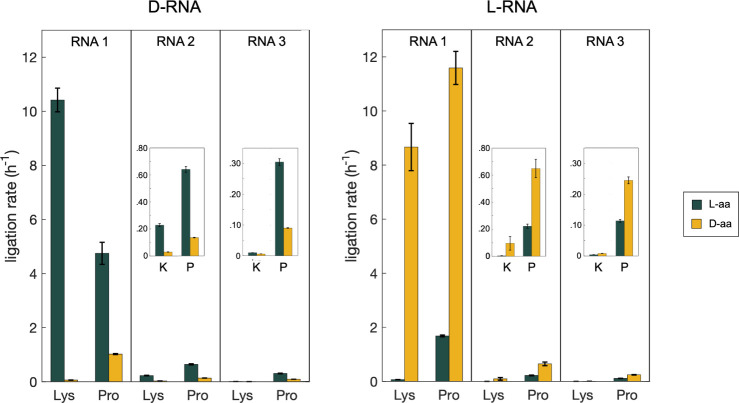
Loop-closing ligation with aminoacylated L-RNA. Rate constants for loop-closing ligation with D-RNA (left) and L-RNA (right). The D- and L- versions of RNA 1 exhibit high stereoselectivity for l- and d-lysine, respectively. Inversion of stereoselectivity is seen for all three D- vs L-RNAs. Each reaction was conducted in three technical replicates at 0 °C in 5 mM MgCl_2_, 100 μM Na_2_EDTA, 100 mM imidazole, and pH 8.0 with 5 μM RNA oligonucleotides. Dark green bars: RNA aminoacylated with L-amino acids; yellow bars: RNA aminoacylated with D-amino acids. Error bars and significance (*p* < 0.05) were estimated using the Monte Carlo method as detailed in the Statistical Analysis section in the Materials and Methods.

**Table 2:** Rate Constants for Loop-Closing Ligation with Aminoacylated L-RNA^a^

		*k*_L_ (h^–1^)	*k*_D_ (h^–1^)	*k*_D_/*k*_L_
RNA 1	Lys	0.067 ± 0.001	8.6 ± 0.9	130
	Pro	1.68 ± 0.03	11.4 ± 0.7	6.8
RNA 2	Lys	0.003 ± 0.001	0.10 ± 0.05	31
	Pro	0.220 ± 0.002	0.65 ± 0.08	2.9
RNA 3	Lys	0.0041 ± 0.0001	0.0082 ± 0.0002	2.0
	Pro	0.114 ± 0.005	0.24 ± 0.01	2.1

a Ligation rates were derived from kinetic models using ligation yields as a function of time and experimental rates for aminoacyl hydrolysis (see Kinetic Analysis section in the Materials and Methods). The rightmost column shows the ratio of kD/kL, and all differences between L- and D-ligation rates are statistically significant (*p* < 0.05, non-parametric test).

## Discussion

We have found a consistent bias across three RNA architectures and with four amino acids for faster loop-closing ligation when D-RNA is aminoacylated with an L-amino acid as opposed to a D-amino acid. As expected from mirror symmetry, when the l-isomer of RNA is aminoacylated, the ligation reaction proceeds preferentially with D-amino acids. Both the reaction rate for loop-closing ligation and the magnitude of the stereoselectivity are highly dependent on the RNA sequence and structure. Interestingly, higher ligation rates are generally associated with higher stereoselectivity. We suggest that a compact folded aminoacyl-RNA structure that is preorganized to constrain the amine of the amino acid in proximity to the activated 5′-phosphate would both enhance the reaction rate and lead to greater stereoselectivity. For example, RNA architecture 1, which displays the highest rates of ligation, also exhibits the highest stereoselectivity (Figure 3). Architecture 1 differs from the other RNA architectures in that it contains a three-nucleotide 5′-overhang and a single-nucleotide 3′-overhang, which is aminoacylated. All four overhanging nucleotides are adenosines, which suggests that the strong stacking interactions of the purine nucleobases could lead to a compact folded structure prior to the loop-closing ligation.^32^ Architecture 2 has a single-nucleotide 5′-overhang and a putative three-nucleotide 3′-overhang; one of the four nucleotides is a pyrimidine, while the other three are purines. This architecture led to slower and less stereoselective loop-closing ligation with all four amino acids for reasons that are unclear. Finally, RNA architecture 3 emerged from a screen for 3′-overhang sequences that led to efficient loop-closing ligation following aminoacylation with glycylimidazolide. Our structural studies^26^ of the glycine-bridged product structure suggest that its 3′-overhang folds so that it largely envelops the glycine. It is therefore less surprising that this RNA exhibits lower rates of loop-closing ligation as well as less stereoselectivity than architectures 1 and 2, since aminoacylation with bulkier amino acids may disrupt the folded RNA structure. We hope that future studies will elucidate the structural basis for stereoselectivity in the loop-closing reactions that we have studied.

In contrast to ligation, hydrolysis rates of aminoacylated RNA did not vary widely with RNA sequence (Tables S1–S4). In all cases, prolyl-RNA hydrolyzed more rapidly than the other aminoacyl-RNA esters, presumably due to the enhanced basicity of the secondary amine. Given that protonation of the amine accelerates hydrolysis of aminoacylated RNA,^33^ we suggest that the differences, though relatively small, in hydrolysis rates between the duplex and single-stranded conditions support the argument that RNA tertiary structure influences the local chemical environment, including the effective pH and steric accessibility, of the aminoacyl amine.

The exclusive use of L-amino acids in biological proteins presents a puzzle. Although a common mechanism that could enrich all 20 amino acids is possible, it has eluded discovery. The concept of chiral propagation is an elegant solution to the problem of the homochirality of proteinogenic amino acids in biology, but the molecular details of this process remain obscure. Ozturk et al.,^20^ following the argument by Tamura and Schimmel,^21^ have posited that the homochirality of RNA was established first, followed by diastereoselective aminoacylation allowing for the synthesis of homochiral peptides from racemic amino acids, and subsequently, chiral transfer processes dictated the handedness of biological metabolites. Alternatively, one might imagine that primordial metabolic reactions catalyzed by ribozymes would begin to fix the chirality of metabolic intermediates, including amino acids, due to the intrinsic stereoselectivity of macromolecular catalysis. However, this leaves unanswered the question of whether there is some underlying reason that D-RNA would ultimately lead to the coded synthesis of peptides and proteins composed exclusively of L-amino acids (and not D-amino acids). Considering the earlier reports from Tamura and Schimmel,^19,21^ Wu et al.,^22^ Roberts et al.,^23^ and Radakovic et al.,^25^ it appears that L-amino acid selectivity arises readily in reactions involving aminoacylated RNA. Our results with loop-closing ligation mediated by aminoacylated RNA confirm and extend this connection between D-RNA and L-amino acids. We have previously demonstrated that loop-closing ligation with aminoacylated RNAs can lead to the assembly of functional ribozymes.^29^ The resulting ribozymes are composed of RNA segments bridged by aminoacyl ester phosphoramidates. We have proposed^29^ that this process, operating in primordial protocells, may have facilitated the assembly of ribozymes from smaller RNA fragments that could have been replicated by nonenzymatic chemistry. Due to the stereoselectivity of loop-closing ligation with aminoacylated RNA, these chimeric ribozymes would largely contain bridging L-amino acids. We hypothesize that in such chimeric ribozymes, the amino acid side chain may have assisted in catalysis, leading to a selection pressure for the emergence of stereoselective aminoacylating ribozymes capable of putting specific amino acids on specific RNAs. The emergence of RNA aminoacylating ribozymes with specificity for L-amino acids would then have provided the evolutionary foundation for the later synthesis of homochiral proteins from L-amino acids. This scheme, while hypothetical, presents an experimentally tractable system for investigating the origin of amino acid homochirality.

## Supplementary Material



## Notes

Materials and Methods, additional experimental details, including supplementary tables and figures that support the results and conclusions in the main text (PDF)

## #

Whitehead Institute, Cambridge, MA 02142

## ∇

Dept. of Biochemistry and Molecular Biology, University of Chicago, Chicago, IL 60637.
